# From parasite to bridge: redefining *Cuscuta* in plant communication networks

**DOI:** 10.1186/s43897-026-00275-2

**Published:** 2026-09-11

**Authors:** Seung Hee Eom, Tae Kyung Hyun

**Affiliations:** Department of Industrial Plant Science and Technology, College of Agriculture, Life and Environment Sciences, Chungbuk National University, Cheongju, 28644 Republic of Korea

Parasitic plants of the genus *Cuscuta* have long been viewed primarily as agricultural pests that exploit host plants for water, nutrients, and photoassimilates. Through specialized haustoria, they establish vascular connections with host tissues and depend extensively on host-derived resources for growth and reproduction (Balios et al. 2025; Clarke et al. 2019; Zagorchev et al. 2025). This resource-centered framework has shaped decades of research and remains the dominant paradigm for interpreting host-parasite interactions. However, recent discoveries indicate that this paradigm is no longer sufficient to explain the biological significance of *Cuscuta*. We argue that *Cuscuta* should now be recognized not merely as a parasite, but as a unique biological bridge that enables the transfer of information between plants. Evidence accumulated over the past decade demonstrates that signals, transcripts, proteins, and developmental regulators move across the host-parasite interface and retain biological activity in recipient tissues (Fichman et al. 2024; Johnson and Axtell 2019; Kim et al. 2014; Shen et al. 2020). These findings challenge the longstanding assumption that haustorial connections function primarily as conduits for resource acquisition. Instead, they reveal that *Cuscuta* creates functional communication pathways through which biologically meaningful information can cross organismal boundaries. Recognizing this role is not simply a refinement of current thinking. It requires a fundamental reassessment of both host-parasite biology and the conceptual boundaries of plant communication.

## Beyond resource acquisition

Three major lines of evidence support this conceptual shift. First, systemic stress signals generated in one host can move through Cuscuta bridges and trigger transcriptional responses in neighboring hosts (Fichman et al. 2024). This discovery demonstrates that biologically meaningful information can be transmitted between otherwise independent plants. Second, extensive molecular trafficking occurs across the host-parasite interface. Thousands of mRNAs move between hosts and *Cuscuta*, and some remain translationally active after transport (Kim et al. 2014; Li et al. 2022; Park et al. 2021). Likewise, parasite-derived microRNAs directly reprogram host defense and hormone-signaling pathways (Hudzik et al. 2023; Johnson and Axtell 2019; Wu et al. 2022). Third, developmental regulators can cross species boundaries. Host-derived FLOWERING LOCUS T proteins move into *Cuscuta* tissues and contribute directly to flowering induction in the parasite (Shen et al. 2020). Individually, these observations may appear as isolated examples of molecular exchange. Collectively, however, they reveal a more profound biological reality. Haustorial connections do not merely redistribute resources. They also establish channels through which information is exchanged, interpreted, and translated into physiological and developmental responses. The traditional resource-acquisition paradigm is therefore no longer sufficient to explain the full biological significance of *Cuscuta*. The emerging evidence instead supports the view that *Cuscuta* functions as a biological bridge linking the physiology of distinct organisms.

## Beyond an organism-centered view of plant signaling

**Fig. 1:**
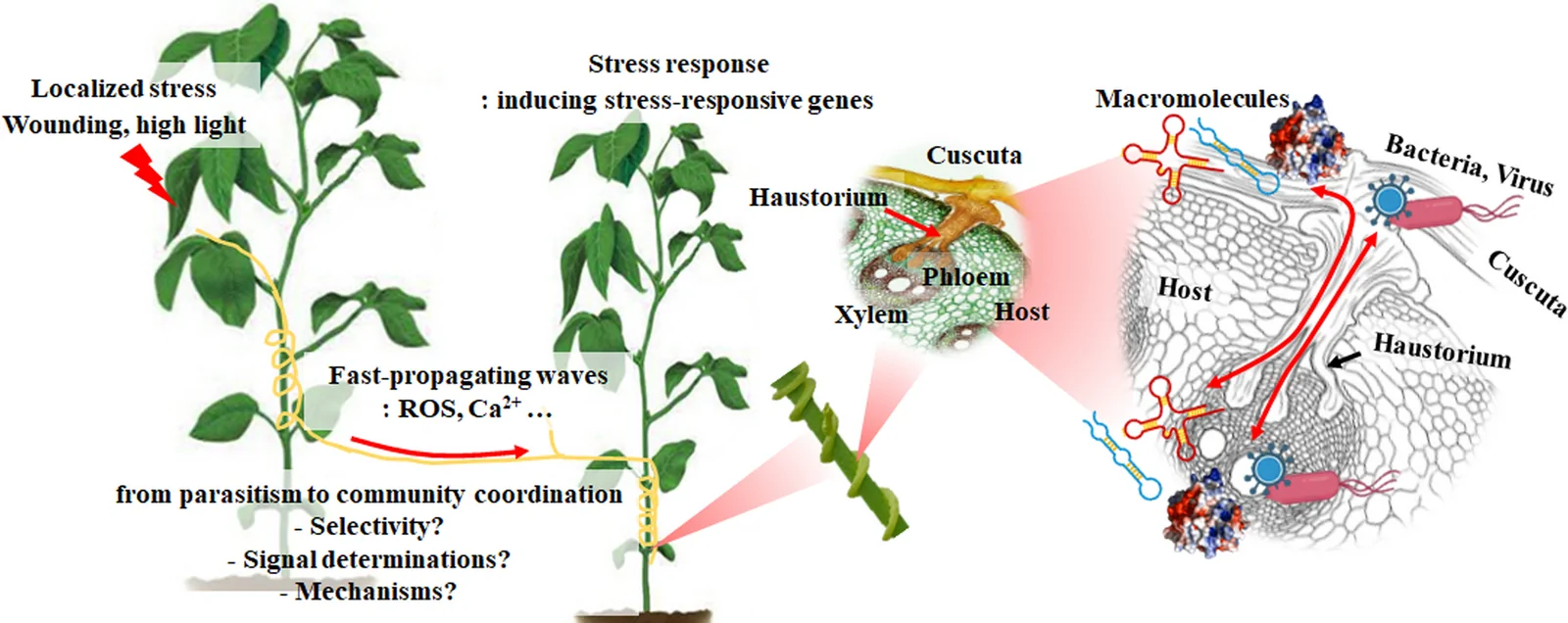
A conceptual model of *Cuscuta* as a biological bridge for inter-plant communication. Through haustorial connections, *Cuscuta* establishes vascular continuity between host plants, creating pathways for the movement of both resources and information. In addition to water, nutrients, and photoassimilates, stress-induced systemic signals, including reactive oxygen species (ROS), calcium signals, and membrane potential changes, can move between connected hosts. Diverse macromolecules, including mRNAs, small RNAs, long non-coding RNAs (lncRNAs), and proteins, also traffic across the host–parasite interface. Collectively, these observations support the view that *Cuscuta* serves as a biological bridge through which biologically meaningful information crosses organismal boundaries, providing a framework for understanding communication within interconnected plant networks

The implications of these findings extend far beyond parasitic plant biology. Most current models of plant signaling are implicitly organism-centered. Whether involving hormones, calcium waves, reactive oxygen species, electrical signals, or mobile RNAs, signaling is generally assumed to originate, propagate, and function within the boundaries of a single plant. The discovery that systemic signals can move between different hosts through *Cuscuta* directly challenges this assumption. We contend that current plant signaling frameworks are insufficient to account for communication across organismal boundaries because they treat information transfer as an organism-bound process. The ability of one plant to influence the physiology of another through vascular connectivity requires a broader conceptual framework in which information transfer is not confined to individual organisms. This shift in perspective is necessary to reinterpret plant communication in interconnected biological systems. Without incorporating communication across organismal boundaries, our understanding of how plants perceive, integrate, and respond to environmental information will remain incomplete. Importantly, we do not suggest that connected plants function as a single integrated superorganism. Rather, *Cuscuta* demonstrates that temporary biological networks can emerge in which information flows among otherwise independent individuals. Similar concepts have been proposed for mycorrhizal networks, but *Cuscuta* provides a uniquely tractable aboveground system for experimentally dissecting these processes. We therefore argue that *Cuscuta* should be recognized as a model system for investigating inter-plant communication and the ecological consequences of biological connectivity Fig. 1.

## Why does this matter for horticulture?

This perspective is particularly relevant for horticulture because current management strategies focus almost exclusively on minimizing the resource costs of parasitism. Such approaches implicitly assume that the primary consequence of *Cuscuta* infestation is nutrient diversion. However, if *Cuscuta* also mediates information transfer among plants, then current frameworks may substantially underestimate its influence on crop performance. Signals transmitted through Cuscuta bridges may alter defense activation, stress responses, developmental timing, and disease susceptibility in connected plants before direct exposure to environmental challenges occurs. For example, if stress-associated signals transmitted through *Cuscuta* prime defense responses in neighboring plants, infestation could influence the spatial distribution of stress resilience across crop populations. Consequently, the effects of infestation may propagate through horticultural systems in ways that cannot be explained by nutrient competition alone. The implications extend to disease management as well. Pathogens and pathogen-associated molecules can move through parasitic connections, but equally important may be the movement of defense-related signals that alter susceptibility across connected plants (Zagorchev et al. 2025). Understanding how information spreads through these biological networks may improve predictions of disease dynamics and inform new strategies for crop protection. Developmental regulation provides another compelling example. The demonstration that flowering signals move from host to parasite establishes that developmental information can cross species boundaries (Shen et al. 2020). Whether analogous mechanisms influence developmental coordination among connected plants remains unknown, but the possibility raises important questions for crop synchronization, productivity, and adaptation. These questions are particularly timely because horticultural systems are increasingly challenged by climate change, emerging diseases, and environmental instability. Under such conditions, overlooking inter-plant information transfer may limit our ability to predict and manage crop responses. Taken together, these observations suggest that the impacts of *Cuscuta* infestation cannot be fully understood within a resource-competition framework. A communication-centered perspective may reveal previously overlooked mechanisms influencing crop performance, resilience, and productivity.

## A research agenda for the next decade

The critical challenge is no longer determining whether information moves through haustorial connections. Existing evidence demonstrates that it does. The priority now is to understand when, how, and why such information transfer matters. Three research directions should receive immediate attention. First, identifying the specific signals and molecular classes that generate functional responses in recipient plants. Second, determining how host identity, environmental conditions, and network structure influence transmission efficiency and signal specificity. Third, establishing whether information transfer alters fitness, stress tolerance, disease dynamics, and crop productivity under realistic agricultural conditions. Addressing these questions will require integrating molecular genetics, imaging, electrophysiology, systems biology, and ecological experimentation. More importantly, it will require moving beyond the outdated assumption that haustorial connections function solely as routes for resource withdrawal. We argue that *Cuscuta* represents one of the most powerful experimental systems currently available for investigating communication across organismal boundaries. Recognizing this role has implications that extend well beyond parasitic plant biology. By redefining *Cuscuta* as a biological bridge rather than simply a parasite, we gain a new framework for understanding how information moves through plant communities and how biological connectivity shapes ecological and horticultural outcomes. More broadly, recognizing *Cuscuta* as a biological bridge compels a re-evaluation of how communication is organized across plant communities and ecological networks.

## Data Availability

Not applicable.

## References

[CR1] Balios VA, Fischer K, Bawin T, Krause K. One organ to infect them all: the Cuscuta haustorium. Ann Bot. 2025;2025(135):823–40. 10.1093/aob/mcae208 PMC12064427 39673400

[CR2] Clarke CR, Timko MP, Yoder JI, Axtell MJ, Westwood JH. Molecular dialog between parasitic plants and their hosts. Annu Rev Phytopathol. 2019;57:279–99. 31226021 10.1146/annurev-phyto-082718-100043

[CR3] Fichman Y, Peláez-Vico MÁ, Mudalige AK, Lee HO, Mittler R, Park SY. Rapid plant-to-plant systemic signaling via a *Cuscuta* bridge. Plant Physiol. 2024;196:716–21. 38888995 10.1093/plphys/kiae339 PMC11483505

[CR4] Hudzik C, Maguire S, Guan S, Held J, Axtell MJ. Trans-species microRNA loci in the parasitic plant *Cuscuta campestris* have a U6-like snRNA promoter. Plant Cell. 2023;35:1834–47. 36896651 10.1093/plcell/koad076 PMC10226579

[CR5] Johnson NR, Axtell MJ. Small RNA warfare: exploring origins and function of trans-species microRNAs from the parasitic plant *Cuscuta*. Curr Opin Plant Biol. 2019;50:76–81. 31029811 10.1016/j.pbi.2019.03.014

[CR6] Kim G, LeBlanc ML, Wafula EK, dePamphilis CW, Westwood JH. Plant science. Genomic-scale exchange of mRNA between a parasitic plant and its hosts. Science. 2014;345:808–11. 25124438 10.1126/science.1253122

[CR7] Li T, Deng Y, Huang J, Liang J, Zheng Y, Xu Q, et al. Bidirectional mRNA transfer between *Cuscuta australis* and its hosts. Front Plant Sci. 2022;13:980033. 36072332 10.3389/fpls.2022.980033 PMC9441868

[CR8] Park SY, Shimizu K, Brown J, Aoki K, Westwood JH. Mobile host mRNAs are translated to protein in the associated parasitic plant *Cuscuta campestris*. Plants. 2021;2021(11):93. 10.3390/plants11010093 PMC8747733 35009096

[CR9] Shen G, Liu N, Zhang J, Xu Y, Baldwin IT, Wu J. *Cuscuta australis* (dodder) parasite eavesdrops on the host plants’ FT signals to flower. Proc Natl Acad Sci U S A. 2020;117:23125–30. 32868415 10.1073/pnas.2009445117 PMC7502711

[CR10] Wu Y, Luo D, Fang L, Zhou Q, Liu W, Liu Z. Bidirectional lncRNA transfer between *Cuscuta* parasites and their host plant. Int J Mol Sci. 2022;23:561. 35008986 10.3390/ijms23010561 PMC8745499

[CR11] Zagorchev L, Zagorcheva T, Teofanova D, Odjakova M. Methods of control of parasitic weeds of the genus *Cuscuta*-current status and future perspectives. Plants (Basel). 2025;14:2321. 40805670 10.3390/plants14152321 PMC12348958

